# Towards the Novel Reasoning among Particles in PSO by the Use of RDF and SPARQL

**DOI:** 10.1155/2014/121782

**Published:** 2014-03-27

**Authors:** Iztok Fister, Xin-She Yang, Karin Ljubič, Dušan Fister, Janez Brest, Iztok Fister

**Affiliations:** ^1^University of Maribor, Faculty of Electrical Engineering and Computer Science, Smetanova 17, 2000 Maribor, Slovenia; ^2^Middlesex University Hendon Campus, London NW4 4BT, UK; ^3^University of Maribor Faculty of Medicine, Taborska 8, 2000 Maribor, Slovenia

## Abstract

The significant development of the Internet has posed some new challenges and many new programming tools have been developed to address such challenges. Today, semantic web is a modern paradigm for representing and accessing knowledge data on the Internet. This paper tries to use the semantic tools such as resource definition framework (RDF) and RDF query language (SPARQL) for the optimization purpose. These tools are combined with particle swarm optimization (PSO) and the selection of the best solutions depends on its fitness. Instead of the local best solution, a neighborhood of solutions for each particle can be defined and used for the calculation of the new position, based on the key ideas from semantic web domain. The preliminary results by optimizing ten benchmark functions showed the promising results and thus this method should be investigated further.

## 1. Introduction

Searching for the optimal solutions of the hardest real-world problems is an active field especially in computer science. An eternal desire of computer scientists is to develop a general problem solver that will be able to cope with all classes of real-world problems. Unfortunately, the most of the so-called clever algorithms are subject of the No Free Lunch Theorem [1]. Regarding this theorem, if one algorithm is good on one class of problems, it does not mean that it will also be good on the other classes of problems. Especially, three domains of algorithms have recently been appeared in the role of general problem solver, as follows: Artificial Intelligence (AI) [2], evolutionary algorithms (EA) [3], and Swarm Intelligence (SI) [4]. While the former mimics operating a human brain, the latter domains are inspired by nature. Evolutionary algorithms are inspired by Darwinian principles of natural evolution [5] according to which the fittest individuals have the greater possibilities for survival and pass on their characteristics to their offspring during a process of reproduction.

Nowadays, evolutionary computation (AC) [6] captures the algorithms involved in evolutionary domain and it considers genetic algorithms (GA) [7], genetic programming [8], evolution strategies (ES) [9], evolutionary programming [10], and differential evolution (DE) [11–13]. The mentioned algorithms differ between each other according to representation of individual. As a result, these kinds of algorithms have been applied to various optimization, modeling, and simulation problems.

However, this paper concentrates on the SI domain that is concerned with the design of multiagent systems with applications, for example, in optimization and in robotics [4]. Inspiration for the design of these systems is taken from the collective behavior of social insects, like ants, termites, and bees, as well as from the behavior of other animal societies, like flocks of birds or schools of fish. Recently, there exist a lot of different algorithms from this domain that is still being developed. Let us mention only the most important members of the SI algorithms, as follows: the particle swarm optimization (PSO) [14], the firefly algorithm (FA) [15, 16], cuckoo search [17], the bat algorithm (BA) [18, 19], and so forth.

The PSO is population-based algorithm that mimics movement of the swarm of particles (e.g., birds) by flying across a landscape, thus searching for food. Each particle in PSO represents the candidate solution of the problem to be solved. Position of the particle consists of the problem parameters that are modified when the virtual particle is moved in the search space. The motion depends on the current particle position and the current position of local best and global best solutions, respectively. The local best solutions denote the best solutions that are whenever arisen on the definite location in the population, while the current best is the best solution whenever found in the whole population. This solution has the main impact on the direction of moving the swarm towards the optimal solution. When this solution is not improved anymore, the population gets stuck into a local optimum.

Mainly, we focused on the new definition of the neighborhood within the PSO algorithm. In place of the local best solutions, the neighborhood is defined using the predefined radius of fitness values around each candidate solution, thus capturing all candidate solutions with the fitness value inside the predefined virtual radius. The size of this neighborhood can be variable. Therefore, at least one but maximum three candidate solutions can be permitted to form this neighborhood. Although this is not the first try how to define the variable neighborhood within the PSO algorithm [20–22], in this paper, such neighborhood is defined using the Resource Description Framework (RDF), SPARQL Protocol, and RDF query language (SPARQL) tools taken from semantic web domain. As a result, the modified RDF-PSO algorithm was developed. Both web tools are appropriate for describing and manipulating decentralized and distributed data. On the other hand, the original PSO algorithm maintains a population of particles that are also decentralized in their nature. An aim of using these web tools was to simulate a distributed population of particles, where each particle is placed on the different location in the Internet.

The remainder of this paper is structured as follows. In Section 2, we outline a short description of the PSO algorithm. The section is finished by introducing the semantic web tools, that is, RDF and SPARQL.  Section 3 concentrates on development of the modified RDF-PSO algorithm.  Section 4 presents the conducted experiments and results obtained with the RDF-PSO algorithms. Finally, Section 5 summarizes our work and potential future directions for the future work are outlined.

## 2. Background

### 2.1. Particle Swarm Optimization

**Figure 1:**
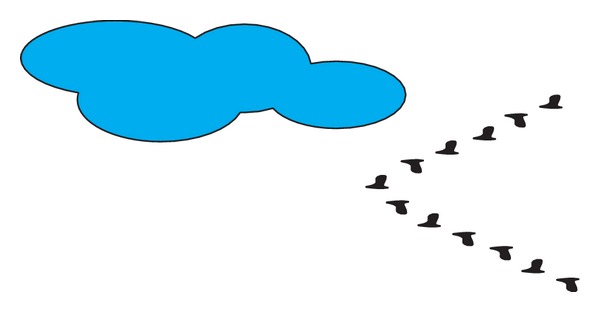
PSO.

Particle swarm optimization (PSO) was one of the first SI algorithms to be presented at an International Conference on Neural Networks by Kennedy and Eberhart in 1995 [14]. PSO is inspired by the social foraging behavior of some animals such as flocking behavior of birds (Figure 1) and schooling behavior of fish [23]. In nature, there are some individuals with better developed instinct for finding food. According to these individuals, the whole swarm is directed into more promising regions in the landscape.

The PSO is a population-based algorithm that consists of *N* particles **x**_*i*_^(*t*)^ = (*x*_*i*1_,…, *x*_*iD*_)^*T*^ representing their position in a *D*-dimensional search space. These particles move across this space with velocity **v**_*i*_^(*t*)^ = (*v*_*i*1_,…, *v*_*iD*_)^*T*^ according to the position of the best particle **x**_best_^(*t*)^ towards the more promising regions of the search space. However, this movement is also dependent on the local best position of each particle **p**_*i*_^(*t*)^ and is mathematically expressed, as follows:

(1) vi(t+1)=vi(t)+c1r1(t)(pi(t)−xi(t)) +c2r2(t)(xbest(t)−xi(t)), for  i=1,…,N,

where *r*_1_^(*t*)^, *r*_2_^(*t*)^ denote the random numbers drawn from the interval [0,1], and *c*_1_, *c*_2_ are constriction coefficients that determine the proportion, with which the local and global best solutions influence the current solution. Then, the new particle position is calculated according to the following expression:

(2) $$\begin{array}{c} x_{i}^{\left(t+1\right)}=x_{i}^{\left(t\right)}+v_{i}^{\left(t\right)},\;\text{for}\;\;i=1,\ldots ,N. \end{array}$$

Pseudocode of the PSO algorithm is illustrated in Algorithm 1.

After finishing the initialization in function “init_particles” (Algorithm 1), the PSO algorithm optimizes a problem by iteratively improving the candidate solution [24, 25]. Thus, two functions are applied. The function “evaluate_the_new_solution” calculates the fitness value of particles obtained after initialization or movement. The movement according to (1) and (2) is implemented in the function “generate_new_solution.”

### 2.2. RDF

The RDF is an XML application devoted to encoding, exchanging, and reusing structural metadata [26]. It enables the knowledge to be represented in symbolical form. Fortunately, this knowledge is human readable. On the other hand, it is understandable to machines. The main characteristic of this framework is that RDF data can be manipulated on decentralized manner and distributed among various servers on the Internet. Resources identified by Uniform Resource Identifier (URI) are described in RDF graphs, where each resource representing the node has many properties that are associated with the resource using the property-type relationship. This relationship represents an edge in RDF graph. Thus, attributes may be atomic in nature (e.g., numbers, text strings, etc.) or represent other resources with their own properties [27].

**Figure 2:**
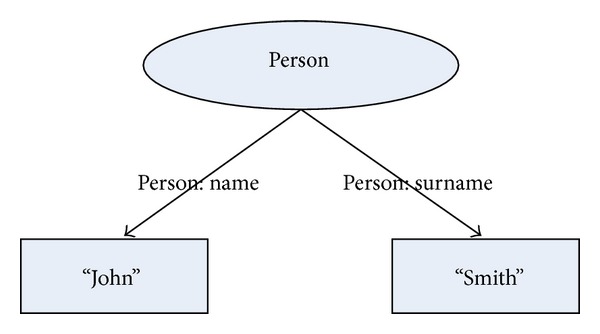
Diagram illustrates a resource Person in RDF graph. The resource consists of two atomic attributes “John” and “Smith” that are assigned to it with relations “PERSON: Name” and “PERSON: Surname”. In other words, the name of the person is “John” and the surname of the same person is “Smith.” Note that the word “PERSON:” denotes a location (URI), where a name space containing the description of semantics for this relation is located.

The resource, property-type relation, and attribute present a triplet suitable for presentation in RDF graph. A sample of this graph is presented in Figure 2, from which two triples (also 2-triples) can be translated from the diagram. These 2-triples are written into a RDF database. In general, the format of this RDF data is serialization of N-triples obtained from the RDF graphs. For instance, the description of RDF database obtained from the RDF graph in Figure 2 is presented in Algorithm 2.

### 2.3. SPARQL

RDF enables data to be decentralized and distributed across the Internet. On the other hand, the SPARQL Protocol has been developed for accessing and discovering RDF data. SPARQL is an RDF query language that has its own syntax very similar to SQL queries. The SPARQL query consists of two parts [28]. The former SELECT clause identifies the variables that appear in the query results, while the latter WHERE clause provides the basic patterns that match against the RDF graph. Usually, these query patterns consist of three parts denoting the resource name, property-type relation, and attribute. As a result, the matched patterns are returned by the query. A sample of SPARQL query is illustrated in Algorithm 3.

As a result of query presented in Algorithm 3, the name “John” and surname “Smith” are returned.

## 3. The Modified RDF-PSO Algorithm

The modified RDF-PSO algorithm implements two features:

- using the variable neighborhood of candidate solutions in place of the local best solutions,
- using the RDF for describing and SPARQL for manipulating this neighborhood.

The main reason for applying these well-known tools from the semantic web domain was to develop a distributed population model that could later be used in other SI algorithms. On the other hand, we try to use the semantic web tools for optimization purposes as well. Fortunately, RDF is suitable tool for describing the distributed population models, in general. In the PSO algorithm, it is applied for describing the relations between particles in population. For our purposes, a relation “is_neigbour_of” is important that each particle determines its neighborhood. Furthermore, SPARQL is used for determining the particles in its neighborhood. As a result, the RDF-PSO algorithm has been established, whose pseudocode is presented in Algorithm 4.

The three main differences distinguish the proposed RDF-PSO with the original PSO algorithm, as follows:

- no local best solutions that are maintained by the RDF-PSO (lines 10–12 omitted in the Algorithm 1),
- defining the neighborhood of candidate solution (line 10 in Algorithm 4),
- generating the new solution according to the defined variable neighborhood relation (line 11 in Algorithm 4).

The relation *𝒩*(**x**_*i*_) = {**x**_*j*_ | **x**_*j*_  is_neighbor_of  **x**_*j*_} (line 10 in Algorithm 4) is defined according to the following relation:

(3) $$\begin{array}{c} if\;\;\text{abs}\left(f\left(x_{j}\right)-f\left(x_{i}\right)\right)\leq R\;then\;\;x_{j}\in 𝒩\left(x_{i}\right), \end{array}$$

where radius *R* defines the necessary maximum fitness distance of two candidate solutions that can be in neighborhood. In fact, this parameter regulates the number of candidate solutions in the neighborhood.

Here, the radius is expressed as $R=\sum_{i=1}^{N}\left|f\left(x_{i}\right)\right|/\sqrt{N}$. Indeed, the neighborhood captures all solutions with the fitness differences less than the radius *R*. Typically, when the radius *R* is small, the size of neighborhood can also be small. However, this assertion holds if the population diversity is higher enough. When the particles are scattered across the search space, no particles are located in the vicinity of each other. Consequently, the size of neighborhood becomes zero. On the other hand, when the particles are crowded around some fitter individuals, the number of its neighbors can be increased enormously. In order to prevent this undersizing and oversizing, the neighborhood size is defined in such a manner that it cannot exceed the value of three and cannot be zero; in other words, |*𝒩*(**x**_*i*_)∈[1,3]|.

For each observed particle **x**_*i*_, the new solution is generated according to the number of neighbors |*𝒩*(**x**_*i*_)| in “generate_new_solution” function. The following modified equation is used in RDF-PSO for calculating the velocity:

(4) vi(t+1)=w·vi(t)+c1r1(t)(xbest(t)−xi(t)) +[∑j=1|𝒩(xj)|cj+1rj+1(t)(pj(t)−xi(t))|𝒩(xi)|],

where, *r*_1_, and *r*_2_ are the real numbers randomly drawn from the interval [0,1], *c*_1_ and *c*_2_ denote the constriction coefficients, **p**_*j*_^(*t*)^ = {**x**_*k*_ | **x**_*k*_  is_neighbor_of  **x**_*i*_∧1 ≤ *k* ≤ *N*}, *j* ∈ [1, |*𝒩*(**x**_*i*_)|], and ∑_*j*=1_^|*𝒩*(**x**_*i*_)|^ *c*_*j*+1_ = 1. Thus, it is expected that the movement of more crowded neighborhood depends on more neighbors. Furthermore, the term between square parenthesis ensures that the proportion of each neighbor as determined by constriction coefficients {*c*_2_, *c*_3_, *c*_4_} never exceeded the value of one.

### 3.1. Representation of a Distributed Population

The rapid growth of the Internet means that new kinds of application architectures have been emerged. The Internet applications are suitable to exploit enormous power of the computers connected to this huge network. Typically, these applications search for data distributed on many servers. These data need to be accessed easily, securely, and efficiently.

This paper proposes the first steps of developing the distributed population model within the PSO algorithm. In line with this, the RDF tool is applied that introduces a description of relations between particles in the population. These relations make us possible to manipulate population members on a higher abstraction level. At the moment, only the relation “is_neighbor_of” is implemented that determines the neighborhood of a specific particle in the population.

**Figure 3:**
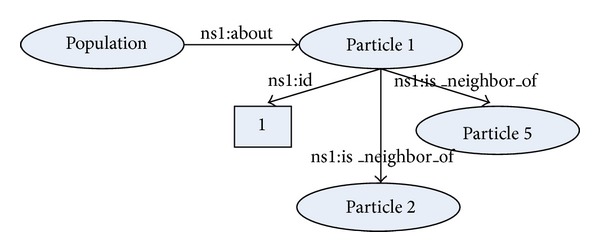
This PSO distributed population model contains the definitions of resources* population* and* particle*. Thus, each* particle* can relate to one or more neighbors and has an identification number. The former represents a reference to other* particles* in a swarm, while the latter is an atomic value.

For this purpose, RDF is devoted for defining the various resources on different Internet servers. In our case, each particle in the population represents the resource that is defined with corresponding property-type relation (e.g., “is_neighbor_of”) and attributes. The RDF graph of the distributed population is illustrated in Figure 3.

The definition of a distributed population in RDF is presented in Algorithm 5, from which it can be seen that two kinds of attributes are encountered in this definition, that is, the references to neighbors of specific particle and its sequence number. Some details are omitted in this algorithm because of the space limitation of this paper. The missing parts of code are denoted by punctuation marks.

### 3.2. Accessing the Distributed Population

The distributed population in RDF can be accessed using the SPARQL query language, whose syntax is similar to the standard SQL syntax. An example of SPARQL query for returning the neighborhood of fourth particle is represented in Algorithm 6. Note that the SPARQL query from the mentioned algorithm will return all attributes that are related to the “resource4” with the relation “is_neighbor_of.”

### 3.3. Implementation Details

The proposed RDF-PSO algorithm was implemented in Python programming language and executed on Linux operating system. Additionally, the following libraries were used:

- *rdflib* which is a python library for working with RDF [29],
- *NumPy* that is the fundamental package for scientific computing with Python [30]
- *matplotlib* that is a python 2D plotting library [31].

The decision for using Python has been taken because there already existed a lot of the PSO implementation. Furthermore, the RDF and SPARQL semantic tools are also supported in this language and ultimately, programming in Python is easy.

## 4. Experiments and Results

The goal of our experimental work was to show that the semantic web tools, that is, RDF and SPARQL can be useful for the optimization purposes as well. Moreover, we want to show that using the variable neighborhood in RDF-PSO can also improve the results of the original PSO.

In line with this, the RDF-PSO algorithm was applied to the optimization of ten benchmark functions taken from literature. The function optimization belongs to a class of continuous optimization problems, where the objective function *f*(**x**) is given and **x** = {*x*_1_,…, *x*_*D*_} is a vector of *D* design variables in a decision space *S*. Each design variable *x*_*i*_ ∈ [*Lb*_*i*_, *Ub*_*i*_] is limited by its lower *Lb*_*i*_ ∈ ℝ and upper *Ub*_*i*_ ∈ ℝ bounds. The task of optimization is to find the minimum of the objective functions.

In the remainder of this section, the benchmark suite is described; then, the experimental setup is presented and finally, the results of experiments are illustrated in detail.

### 4.1. Test Suite

The test suite consisted of ten functions, which were selected from the literature. However, the primary reference is the paper by Yang [32] that proposed a set of optimization functions suitable for testing the newly developed algorithms. The definitions of the benchmark functions are represented in Table 1, while their properties are illustrated in Table 2.

Table 2 consists of five columns that contain the function identifications (tag *f*), their global optimum (tag f*), the values of optimal design variables (tag x*), the lower and upper bounds of the design variables (tag* Bound*), and their characteristics (tag* Characteristics*). The lower and upper bounds of the design variables determine intervals that limit the size of the search space. The wider is the interval, the wider is the search space. Note that the intervals were selected, so that the search space was wider than those proposed in the standard literature. The functions within the benchmark suite can be divided into* unimodal* and* multimodal*. The multimodal functions have two or more local optima. Typically, the multimodal functions are more difficult to solve. The most complex functions are those that have an exponential number of local optima randomly distributed within the search space.

### 4.2. Experimental Setup

This experimental study compares the results of the RDF-PSO using different kind of distributed populations within the original PSO algorithm. All PSO algorithms used the following setup. The parameter *w* was randomly drawn from the interval [0.4,0.9], while the constriction coefficients were set as *c*_1_ = *c*_2_ = 1.0. As a termination condition, the number of fitness function evaluations was considered. It was set to FEs = 1000 · *D*, where *D* denotes dimension of the problem. In this study, three different dimensions of functions were applied; that is, *D* = 10, *D* = 30, and *D* = 50. However, the population size is a crucial parameter for all population-based algorithms that have a great influence on their performance. In line with this, extensive experiments had been run in order to determine the most appropriate setting of this parameter by all algorithms in the test. As a result, the most appropriate setting of this parameter *N* = 100 was considered for the experiments. Parameters, like the termination condition, dimensions of the observed functions, and the population size were also used by the other algorithms in experiments.

The PSO algorithms are stochastic in nature. Therefore, statistical measures, like minimum, maximum, average, standard deviation, and median, were accumulated after 25 runs of the algorithms in order to fairly estimate the quality of solutions.

### 4.3. Results

The comparative study was conducted in which we would like to show, firstly, that the semantic web tools can be successfully applied to the optimization purposes as well and, secondly, that using the distributed population affects the results of the original PSO algorithm. In the remainder of this section, a detailed analysis of RDF-PSO algorithms is presented.

#### 4.3.1. Analysis of the RDF-PSO Algorithms

In this experiment, the characteristics of the RDF-PSO algorithm were analyzed. In line with this, the RDF-PSO with neighborhood size of one (RDF1), the RDF-PSO with neighborhood size of two (RDF2), and the RDF-PSO with neighborhood size of tree (RDF3) were compared with the original PSO algorithm (PSO) by optimizing ten benchmark functions with dimensions *D* = 10, *D* = 30, and *D* = 50. The obtained results by the optimization of functions with dimension *D* = 30 are aggregated in Table 3. Note that the best average values are for each function presented bold in the table.

From Table 3, it can be seen that the best average values were obtained by the RDF-1 algorithm eight times, that is, by *f*_1_ − *f*_4_, *f*_6_, *f*_8_, and *f*_10_. The best results were two times observed also by the original PSO algorithm, that is, *f*_5_ and *f*_9_. On average, the results of the other two RDF-PSO algorithms, that are, RDF-2 and RDF-3, were better than the results of the original PSO algorithm.

In order to statistically estimate the quality of solution, the Friedman nonparametric test was conducted. Each algorithm enters this test with five statistical measures for each of observed functions. As a result, each statistical classifier (i.e., various algorithms) consists of 5 · 10 = 50 different variables. The Friedman test [33, 34] compares the average ranks of the algorithms. The closer the rank to one, the better is the algorithm in this application. A null hypothesis states that two algorithms are equivalent and, therefore, their ranks should be equal. If the null hypothesis is rejected, that is, the performance of the algorithms is statistically different, the Bonferroni-Dunn test [35] is performed that calculates the critical difference between the average ranks of those two algorithms. When the statistical difference is higher than the critical difference, the algorithms are significantly different. The equation for the calculation of critical difference can be found in [35].

**Figure 4:**
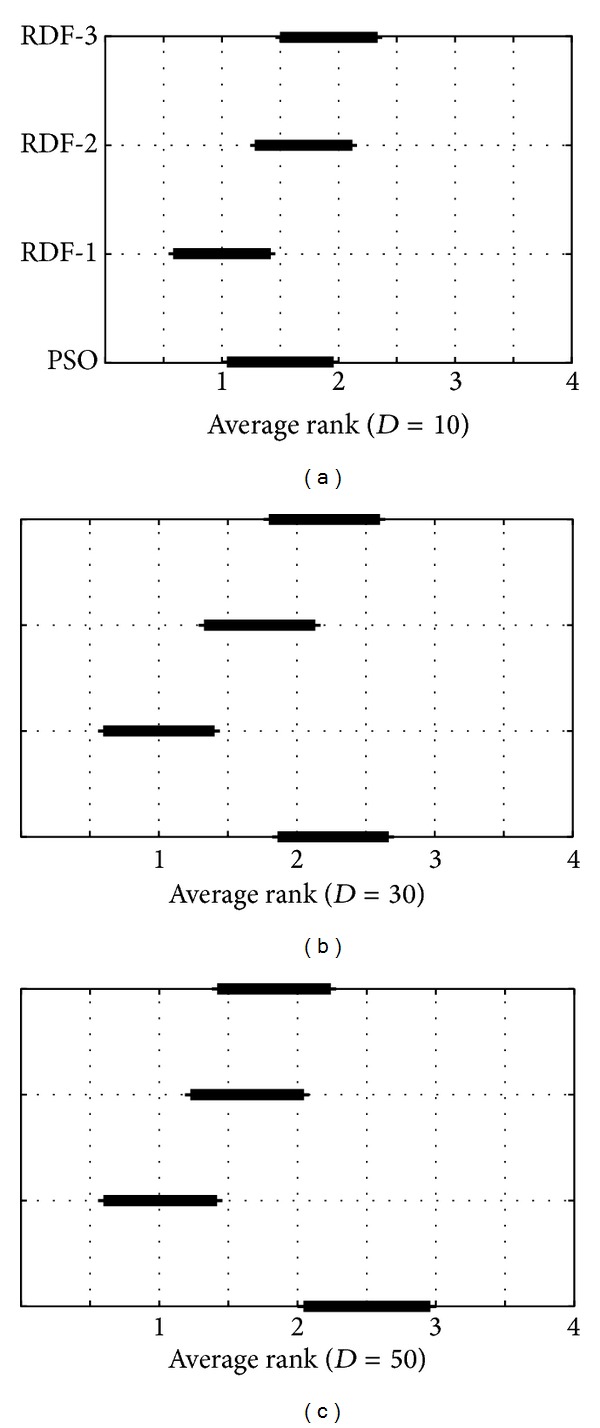
Results of the Friedman nonparametric test.

Friedman tests were performed using the significance level 0.05. The results of the Friedman nonparametric test are presented in Figure 4 where the three diagrams show the ranks and confidence intervals (critical differences) for the algorithms under consideration. The diagrams are organized according to the dimensions of functions. Two algorithms are significantly different if their intervals do not overlap.

The first diagram in Figure 4 shows that the RDF-1 algorithm significantly outperforms the RDF-3 algorithm. Interestingly, the results of the original PSO are also better than the results of the RDF-2 and RDF-3 algorithm. The situation is changed in the second (by *D* = 30) and third diagram (by *D* = 50), where RDF-3 improves the results of the RDF-3 and the original PSO, but not the RDF-2 algorithm. Additionally, the RDF-2 is significantly better than the original PSO also by *D* = 50.

In summary, the RDF-1 exposes the best results between all the other algorithms in tests by all observed dimensions of functions. On the other hand, the original PSO algorithm is only comparable with the modified PSO algorithms by optimizing the low dimensional functions (*D* = 10). The question why the RDF-PSO with neighborhood size of one outperformed the other RDF-PSO algorithms remains open for the future work. At this moment, it seems that here the primary role plays the constriction coefficients that determine an influence of specific neighbors.

## 5. Conclusion

The aim of this paper was twofold. First is to prove that the semantic web tools, like RDF and SPARQL, can also be used for the optimization purposes. Second is to show that the results of the modified RDF-PSO using the variable neighborhood are comparable with the results of the original PSO algorithm.

In line with the first hypothesis, a distributed population model was developed within the PSO algorithm that is suitable for describing the variable neighborhood of particles in the population. Furthermore, moving particles across the search space depends on all the particles in the neighborhood in place of the local best solutions as proposed in the original PSO algorithm.

In order to confirm the second hypothesis, the benchmark suite of ten well-known functions from the literature was defined. The results of extensive experiments by optimization of benchmark functions showed that the optimal neighborhood size within the RDF-PSO algorithm is one (RDF1). This variant of the RDF-PSO also outperformed the original PSO algorithm.

The distributed population model extends the concept of population in SI. This means that the population is no longer a passive data structure for storing particles. Not only can the particles now be distributed, but also some relations can be placed between the population members. In this proof of concept, only one relation was defined, that is, “is_neighbor_of.” Additionally, not the whole definition of the distributed population was put onto Internet at this moment. Although we are at the beginning of the path of how to make an intelligent particle in swarm intelligence algorithms, the preliminary results are encouraging and future researches would investigate this idea of distributed population models in greater detail.

## Figures and Tables

**Algorithm 1:**
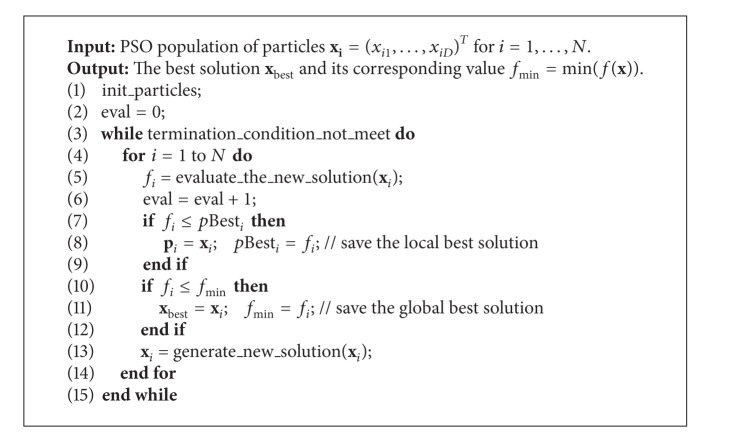
Pseudocode of the classic PSO algorithm.

**Algorithm 2:**
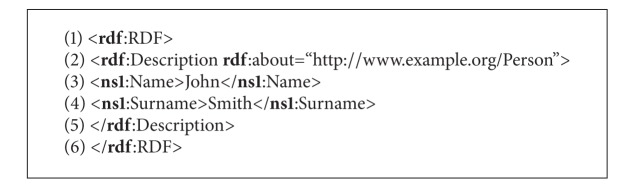
Pseudocode of the Person description in RDF.

**Algorithm 3:**
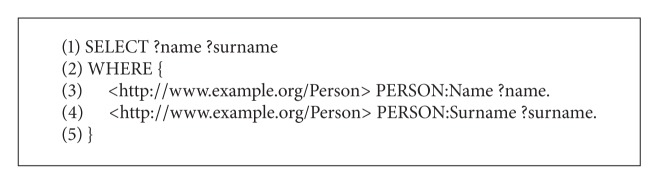
Example of SPARQL query.

**Algorithm 4:**
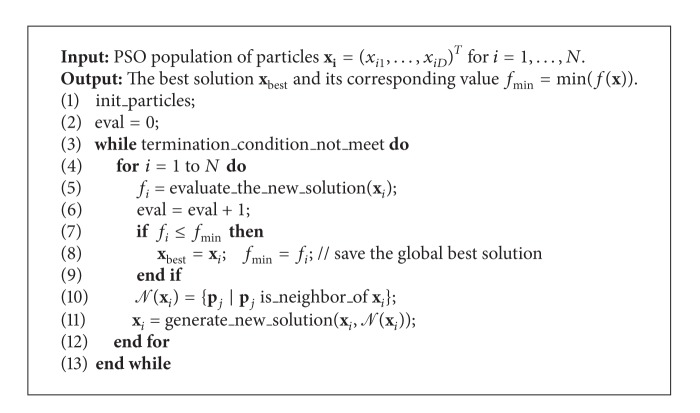
The proposed RDF-PSO algorithm.

**Algorithm 5:**
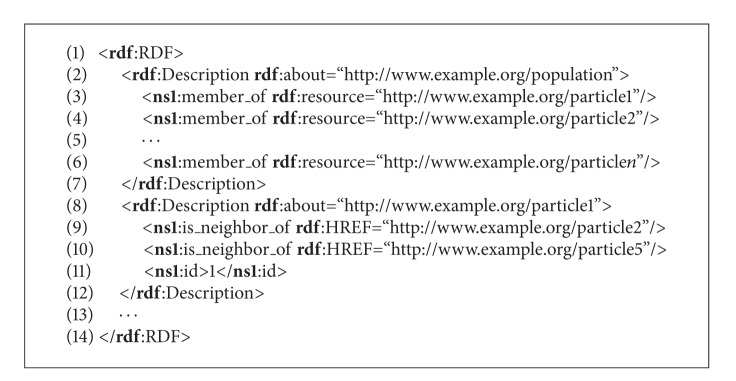
Pseudocode of the PSO population in RDF.

**Algorithm 6:**
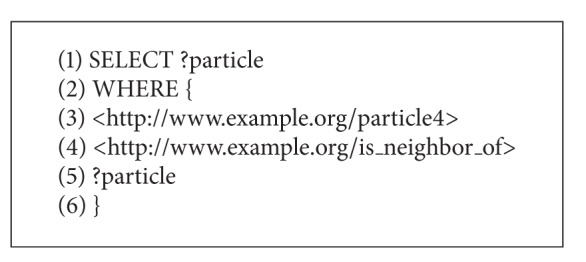
SPAQL query that returns particles in the neighborhood of particle4.

**Table 1:** Definitions of benchmark functions.

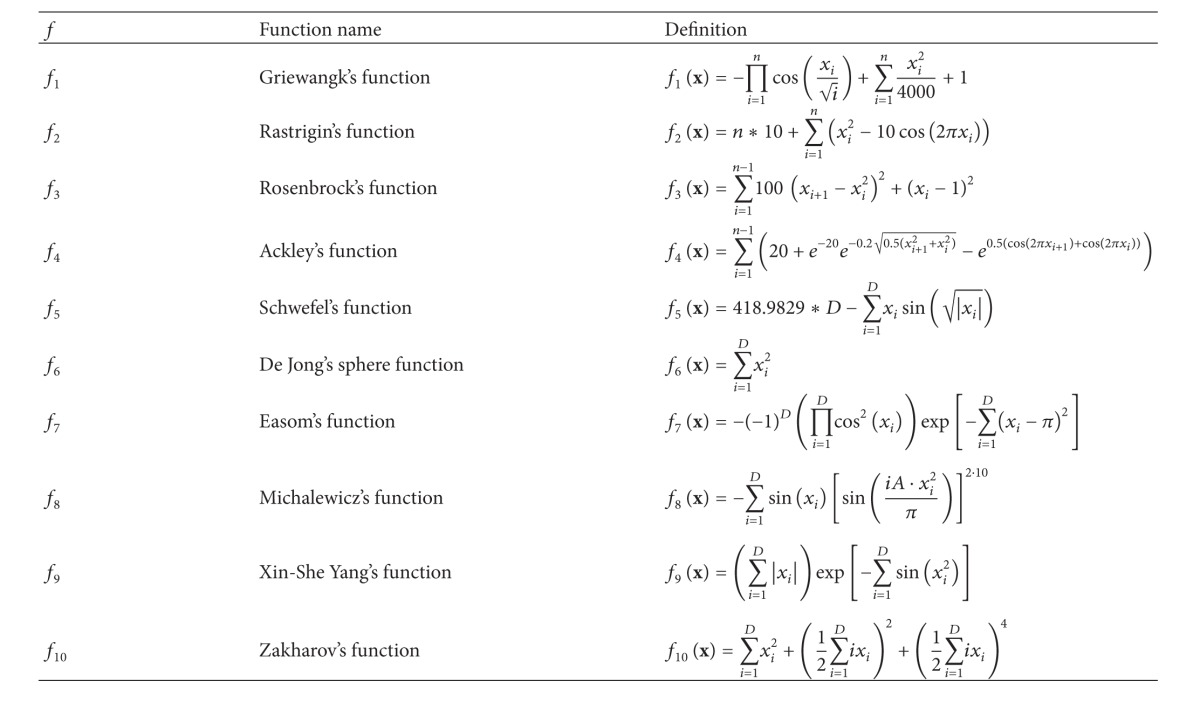

**Table 2:** Properties of benchmark functions.

*f*	f*	x*	Bounds	Characteristics
*f*_1_	0.0000	(0,0,…, 0)	[−600,600]	Highly multi-modal
*f*_2_	0.0000	(0,0,…, 0)	[−15,15]	Highly multi-modal
*f*_3_	0.0000	(1,1,…, 1)	[−15,15]	Multiple local optima
*f*_4_	0.0000	(0,0,…, 0)	[−32.768,32.768]	Highly multi-modal
*f*_5_	0.0000	(0,0,…, 0)	[−500,500]	Highly multi-modal
*f*_6_	0.0000	(0,0,…, 0)	[−600,600]	Uni-modal, convex
*f*_7_	−1.0000	(*π*, *π*,…, *π*)	[−2*π*, 2*π*]	Multiple local optima
*f*_8_	−1.8013^1^	(2.20319,1.57049)^1^	[0, *π*]	Multiple local optima
*f*_9_	0.0000	(0,0,…, 0)	[−2*π*, 2*π*]	Multiple local optima
*f*_10_	0.0000	(0,0,…, 0)	[−5,10]	Uni-modal

^1^These values are valid for dimensions *D* = 2.

**Table 3:** Comparing the results of different PSO algorithms (*D* = 30).

Alg.	Meas.	*f*_1_	*f*_2_	*f*_3_	*f*_4_	*f*_5_
PSO	Best	7.40*E* − 001	5.83*E* + 002	7.08*E* + 004	2.00*E* + 001	1.55*E* + 003
	Worst	1.41*E* + 000	5.65*E* + 003	1.10*E* + 007	2.06*E* + 001	9.77*E* + 003
	Mean	1.06*E* + 000	1.44*E* + 003	3.22*E* + 006	2.03*E* + 001	5.16**E** + 003
	StDev	1.06*E* + 000	1.13*E* + 003	2.04*E* + 006	2.04*E* + 001	7.44*E* + 003
	Mean	1.37*E* − 001	1.02*E* + 003	3.14*E* + 006	1.90*E* − 001	5.97*E* + 003

RDF-1	Best	8.87*E* − 014	4.55*E* − 010	2.85*E* + 001	5.31*E* − 005	8.77*E* + 003
	Worst	3.33*E* − 010	1.95*E* − 006	2.88*E* + 001	9.64*E* − 003	1.05*E* + 004
	Mean	3.40**E** − 011	2.18**E** − 007	2.87**E** + 001	2.61**E** − 003	9.88*E* + 003
	StDev	1.27*E* − 011	4.81*E* − 008	2.87*E* + 001	1.53*E* − 003	9.95*E* + 003
	Median	6.91*E* − 011	4.90*E* − 007	5.85*E* − 002	2.68*E* − 003	3.99*E* + 002

RDF-2	Best	1.10*E* − 005	3.37*E* + 000	3.95*E* + 001	2.78*E* − 001	8.77*E* + 003
	Worst	2.42*E* − 001	7.03*E* + 001	3.42*E* + 002	3.64*E* + 000	1.04*E* + 004
	Mean	6.03*E* − 002	3.08*E* + 001	1.64*E* + 002	1.95*E* + 000	9.73*E* + 003
	StDev	4.12*E* − 002	2.52*E* + 001	1.36*E* + 002	1.72*E* + 000	9.65*E* + 003
	Mean	5.80*E* − 002	2.12*E* + 001	9.62*E* + 001	1.04*E* + 000	4.43*E* + 002

RDF-3	Best	3.07*E* − 005	1.85*E* + 001	5.49*E* + 001	9.11*E* − 001	8.81*E* + 003
	Worst	2.52*E* − 001	1.56*E* + 002	4.03*E* + 002	4.56*E* + 000	1.03*E* + 004
	Mean	8.94*E* − 002	7.75*E* + 001	1.77*E* + 002	2.63*E* + 000	9.70*E* + 003
	StDev	7.62*E* − 002	7.60*E* + 001	1.64*E* + 002	2.66*E* + 000	9.78*E* + 003
	Mean	5.51*E* − 002	3.46*E* + 001	8.38*E* + 001	1.11*E* + 000	4.19*E* + 002

Evals	Meas.	*f*_6_	*f*_7_	*f*_8_	*f*_9_	*f*_10_

PSO	Best	1.10*E* − 003	0.00*E* + 000	− 1.77*E* + 001	6.94*E* − 012	5.39*E* − 001
	Worst	3.75*E* − 001	0.00*E* + 000	− 8.45*E* + 000	5.38*E* − 011	2.42*E* + 001
	Mean	1.13*E* − 001	0.00*E* + 000	− 1.42*E* + 001	1.30**E** − 011	5.26*E* + 000
	StDev	6.89*E* − 002	0.00*E* + 000	− 1.41*E* + 001	8.60*E* − 012	3.36*E* + 000
	Mean	1.24*E* − 001	0.00*E* + 000	1.87*E* + 000	1.05*E* − 011	5.35*E* + 000

RDF-1	Best	7.80*E* − 014	0.00*E* + 000	− 6.45*E* + 000	1.83*E* − 007	2.47*E* − 012
	Worst	3.55*E* − 009	0.00*E* + 000	− 3.85*E* + 000	1.39*E* − 005	5.70*E* − 007
	Mean	3.83**E** − 010	0.00*E* + 000	− 4.81**E** + 000	3.68*E* − 006	2.59**E** − 008
	StDev	2.97*E* − 011	0.00*E* + 000	− 4.74*E* + 000	3.35*E* − 006	1.34*E* − 009
	Mean	8.15*E* − 010	0.00*E* + 000	6.12*E* − 001	3.29*E* − 006	1.13*E* − 007

RDF-2	Best	6.63*E* − 004	0.00*E* + 000	− 6.47*E* + 000	3.57*E* − 009	2.55*E* − 001
	Worst	2.03*E* + 000	0.00*E* + 000	− 4.06*E* + 000	1.73*E* − 007	1.99*E* + 002
	Mean	4.36*E* − 001	0.00*E* + 000	− 4.93*E* + 000	6.03*E* − 008	2.54*E* + 001
	StDev	1.48*E* − 001	0.00*E* + 000	− 4.81*E* + 000	3.83*E* − 008	5.92*E* + 000
	Mean	5.58*E* − 001	0.00*E* + 000	5.92*E* − 001	4.64*E* − 008	4.90*E* + 001

RDF-3	Best	5.21*E* − 003	0.00*E* + 000	− 6.27*E* + 000	2.42*E* − 009	5.94*E* + 000
	Worst	2.09*E* + 000	0.00*E* + 000	− 4.09*E* + 000	1.31*E* − 007	4.53*E* + 002
	Mean	8.47*E* − 001	0.00*E* + 000	− 5.03*E* + 000	3.82*E* − 008	1.09*E* + 002
	StDev	8.14*E* − 001	0.00*E* + 000	− 5.05*E* + 000	3.35*E* − 008	6.65*E* + 001
	Mean	5.73*E* − 001	0.00*E* + 000	5.92*E* − 001	3.08*E* − 008	1.15*E* + 002

## References

[1] Wolpert DH, Macready WG (1997). No free lunch theorems for optimization. *IEEE Transactions on Evolutionary Computation*.

[2] Russell S, Norvig P (2009). *Artificial Intelligence: A Modern Approach*.

[3] Eiben AE, Smith JE (2003). *Introduction to Evolutionary Computing*.

[4] Blum C, Merkle D (2008). *Swarm Intelligence: Introduction and Applications*.

[5] Darwin C (1859). *The Origin of Species*.

[6] Paszkowicz W (2009). Genetic algorithms, a nature-inspired tool: survey of applications in materials science and related fields. *Materials and Manufacturing Processes*.

[7] Goldberg D (1996). *Genetic Algorithms in Search, Optimization, and Machine Learning*.

[8] Koza J (1994). *Genetic Programming 2—Automatic Discovery of Reusable Programs*.

[9] Bck T (1996). *Evolutionary Algorithms in Theory and Practice—Evolution Strategies, Evolutionary Programming, Genetic Algorithms*.

[10] Fogel L, Owens A, Walsh M (1996). *Artificial Intelligence through Simulated Evolution*.

[11] Storn R, Price K (1997). Differential evolution—a simple and efficient heuristic for global optimization over continuous spaces. *Journal of Global Optimization*.

[12] Brest J, Greiner S, Bošković B, Mernik M, Žumer V (2006). Self-adapting control parameters in differential evolution: a comparative study on numerical benchmark problems. *IEEE Transactions on Evolutionary Computation*.

[13] Das S, Suganthan PN (2011). Differential evolution: a survey of the state-of-the-art. *IEEE Transactions on Evolutionary Computation*.

[14] Kennedy J, Eberhart R Particle swarm optimization.

[15] Fister I, Fister I, Yang X-S, Brest J (2013). A comprehensive review of firefly algorithms. *Swarm and Evolutionary Computation*.

[16] Fister I, Yang X-S, Brest J, Fister I (2013). Modified firefly algorithm using quaternion representation. *Expert Systems with Applications*.

[17] Fister Jr. I, Fister D, Fister I (2013). A comprehensive review of cuckoo search: variants and hybrids. *International Journal of Mathematical Modelling and Numerical Optimisation*.

[18] Yang X-S (2010). A new metaheuristic bat-inspired algorithm. *Nature Inspired Cooperative Strategies for Optimization (NICSO 2010)*.

[19] Fister Jr. I, Fister D, Fister I Differential evolution strategies with random forest regression in the bat algorithm.

[20] Suganthan PN Particle swarm optimiser with neighbourhood operator.

[21] Liu H, Abraham A, Choi O, Moon SH (2006). Variable neighborhood particle swarm optimization for multi-objective flexible job-shop scheduling problems. *Simulated Evolution and Learning*.

[22] Kennedy J (2010). Particle swarm optimization. *Encyclopedia of Machine Learning*.

[23] Chakraborti N, Jayakanth R, Das S, Çalişir ED, Erkoç Ş (2007). Evolutionary and genetic algorithms applied to Li+-C system: calculations using differential evolution and particle swarm algorithm. *Journal of Phase Equilibria and Diffusion*.

[24] Eberhart RC, Shi Y Particle swarm optimization: developments, applications and resources.

[25] Shi Y, Eberhart R Modified particle swarm optimizer.

[26] Miller E (1998). An introduction to the resource description framework. *D-Lib Magazine*.

[27] Allemang D, Hendler J (2011). *Semantic Web for the Working Ontologist: Effective Modeling in RDFS and OWL*.

[28] Harris S, Seaborne A (2013). *Sparql 1.1 Query Language*.

[29] http://code.google.com/p/rdflib/.

[30] Numpy http://www.numpy.org/.

[31] Matplotlib http://matplotlib.org/.

[32] Yang X-S, Yang X-S (2010). Appendix A: test problems in optimization. *Engineering Optimization*.

[33] Friedman M (1937). The use of ranks to avoid the assumption of normality implicit in the analysis of variance. *Journal of the American Statistical Association*.

[34] Friedman M (1940). A comparison of alternative tests of significance for the problem of *m* rankings. *The Annals of Mathematical Statistics*.

[35] Demšar J (2006). Statistical comparisons of classifiers over multiple data sets. *Journal of Machine Learning Research*.

